# Evaluating the Operational Efficiency and Quality of Tertiary Hospitals in Taiwan: The Application of the EBITDA Indicator to the DEA Method and TOBIT Regression

**DOI:** 10.3390/healthcare10010058

**Published:** 2021-12-29

**Authors:** Chung-Shun Lin, Cheng-Ming Chiu, Yi-Chia Huang, Hui-Chu Lang, Ming-Shu Chen

**Affiliations:** 1 Department of Accounting, Far Eastern Memorial Hospital, New Taipei City 220, Taiwan; hermes@mail.femh.org.tw; 2 Department of Rehabilitation, Far Eastern Memorial Hospital, New Taipei City 220, Taiwan; femh71025@femh.org.tw; 3 Institute of Hospital and Health Care Administration, National Yang Ming Chiao Tung University, Taipei City 112, Taiwan; tina30710001@gm.ym.edu.tw; 4 Department of Healthcare Administration, College of Healthcare & Management, Asia Eastern University of Science and Technology, New Taipei City 220, Taiwan

**Keywords:** tertiary hospital, operational effectiveness, data envelopment analysis (DEA), Tobit regression, earnings before interest, taxes, depreciation and amortization (EBITDA)

## Abstract

This study estimates the efficiency of 19 tertiary hospitals in Taiwan using a two-stage analysis of Data Envelopment Analysis (DEA) and TOBIT regression. It is a retrospective panel-data study and includes all the tertiary hospitals in Taiwan. The data were sourced from open information hospitals legally required to disclose to the National Health Insurance (NHI) Administration, Ministry of Health and Welfare. The variables, including five inputs (total hospital beds, total physicians, gross equipment, fixed assets net value, the rate of emergency transfer in-patient stay over 48 h) and six outputs (surplus or deficit of appropriation, length of stay, the total relative value units [RVUs] for outpatient services, total RVUs for inpatient services, self-pay income, modified EBITDA) were adopted into the Charnes, Cooper and Rhodes (CCR) and Banker, Charnes and Cooper (BCC) model. In the CCR model, the technical efficiency (TE) from 2015–2018 increases annually, and the average efficiency of all tertiary hospitals is 96.0%. In the BCC model, the highest pure technical efficiency (PTE) was in 2018 and the average efficiency of all medical centers is 99.1%. The average scale efficiency of all medical centers was 96.8% in the BBC model, meaning investment can be reduced by 3.2% and the current production level can be maintained with a fixed return to scale. Correlation coefficient analysis shows that all variables are correlated positively; the highest was the number of beds and the number of days in hospital (r = 0.988). The results show that TE in the CCR model was similar to PTE in the BCC model in four years. The difference analysis shows that more hospitals must improve regarding surplus or deficit of appropriation, modified EBITDA, and self-pay income. TOBIT regression reveals that the higher the bed-occupancy rate and turnover rate of fixed assets, the higher the TE; and the higher number of hospital beds per 100,000 people and turnover rate of fixed assets, the higher the PTE. DEA and TOBIT regression are used to analyze the other factors that affect medical center efficiency, and different categories of hospitals are chosen to assess whether different years or different types of medical centers affect operational performance. This study provides reference values for the improvable directions of relevant large hospitals’ inefficiency decision-making units through reference group analysis and slack variable analysis.

## 1. Introduction

The tertiary hospital, also known as a medical center, is the highest-level teaching hospital in Taiwan. It plays the main role in medical care for emergency, critical, and intractable illnesses. In addition to shouldering significant clinical medical capacity, it must also carry the responsibilities of medical research, teaching, and training. Moreover, it is the principal institution for implementing national health and welfare policies [1], medical centers currently possess 24% of the total hospital beds in Taiwan and employ 44% of Taiwan’s physicians and 34% of its non-physician medical personnel. They use these resources to provide 27% of the in-patient days of Taiwan. The attributes of tertiary hospitals in Taiwan are divided into two categories: public and private hospitals, and include different types such as government, foundation, religious, and university-affiliated hospitals. This may lead to differences in the efficiency of resource application and financial operation between different hospitals, which may in turn result in variabilities in hospitals’ operational performance. Facing the triple pressures of health insurance finance, patient care, and internal operations, medical institutions must actively seek more efficient operational methods. Where there are limited medical resources, medical institutions hope to obtain excellent service volume, financial indicators, and medical quality indicators (output) with minimum resources, including health care workers, hospital beds, or equipment (input) [2]. Only in this manner can medical centers achieve economies of scale and optimize operational efficiency. There are currently 19 tertiary hospitals in Taiwan. Previous research on the effectiveness of medical institutions has mostly included comparisons between public and private hospitals or between hospitals of different levels [2,3,4,5], and there have been few studies which have compared the operational performance between tertiary hospitals with different attributes or categories. Because hospitals of different levels had different cost structures or states of operation, a cost–benefit analysis that merely compares public and private hospitals without considering hospitals of different levels or with different attributes could possibly yield analysis results that are of low value for large medical centers in the application. Therefore, this study has reference value for the comparison of large medical centers. To evaluate the operational performance of an institution or organization, the use of Data Envelopment Analysis (DEA) as one of the methods to evaluate performance can provide an objective measurement standard, assess the current operating status of the organization, and provide managers with directions and possibilities for improvement. This can also guide the organization to continue to work toward their goals. Hence, effective performance evaluation has positive benefits for the organization and can promote its development. A medical institution is a very special kind of organization, especially the medical centers that belong to the highest-level medical institutions in Taiwan. Even though almost all of these medical institutions are non-profit organizations, profitability remains a variable that affects a medical institution’s sustainability. On this basis, this study introduced a medical institution’s modified earnings before interest, taxes, depreciation, and amortization (EBITDA) as an output variable in DEA. Whether it is a public, school-affiliated, or foundation hospital, each medical center must also assume social responsibilities and assist in promoting national health and welfare policies, and even includes diplomatic international medical services. (R2-Q3.4) If there are any operational problems, the impact on the general public and the country will be extremely significant. Therefore, the operational performance of such hospitals should be carefully evaluated at all times.

There are many ways to evaluate performance, and the mathematical models used are also different. Sherman revealed that the most commonly used methods for assessing efficiency are as follows: ratio analysis, balanced score card (BSC), total factor productivity (TFP), regression analysis, production frontier approach (PFA), and DEA in 1984 [6]. Ratio analysis can only evaluate a single input and a single output factor; while BSC, TFP, and PFA are suitable for problems involving multiple inputs and a single output. DEA can simultaneously evaluate multiple investment and output factors, making it more suitable for applications in the complex environment of medical institutions and the healthcare industry. In the past, regression analysis was the most commonly used statistical method for evaluating the effectiveness of medical institutions. BSC and DEA have more often been used in recent studies. BSC is a performance management tool developed by Kaplan and Norton in 1992 [7]. It is mainly composed of four perspectives; respectively, the financial perspective, customer perspective, internal perspective, and learning and growth perspective. With experts assigning scores, the organization’s development in all aspects can be balanced. The DEA method is a concept of measuring efficiency with the efficiency frontier, as proposed by Farrell in 1957 [8]. The linear programming (LP) model is used to acquire the efficiency frontier, which belongs to the same kind of linear programming as regression analysis. DEA is a non-parametric analysis method (non-parametric approach) that uses linear programming to establish the efficiency frontier of all observation data. Organizations or units that estimate efficiency for decision-making are called decision-making units (DMUs). Their advantage is that they can be applied to the efficiency analysis of multiple input and output variables, and can evaluate the overall efficiency of a unit or organization. Furthermore, the weights of the input and output items are obtained through calculations and are thus unaffected by human subjectivity. The disadvantage is that the efficiency obtained by DEA is merely the relative efficiency between the DMUs and cannot be extrapolated for comparison. DEA can evaluate the operational performance of multiple DMUs simultaneously. It uses a non-parametric parameter analysis and can be used in multiple inputs and outputs to obtain a comparable relative efficiency value. In recent years, it has been widely used in the performance evaluation of non-profit enterprises. At present, it is an analytical method commonly used to measure medical performance in health-care-related fields [9].

Nunamaker (1983) used DEA in the medical field for the first time, analyzing the relative efficiencies of nursing services in 17 hospitals using inpatient expenditure as the input variable and the three items of inpatient days for the elderly and children, female patients, and other types of patients as output variables [10]. In 1984, Sherman first applied DEA to the hospital level for efficiency evaluation, using the number of physicians and surgeons, the working hours of full-time staff, the cost of medical materials, and the year-round total number of hospital beds as input variables, and the number of inpatient days for elderly with health insurance coverage, the inpatient days for elderly without health insurance coverage, the number of intern nurses, and the number of intern residents as output variables [6]. Since then, the number of DEA-related publications has grown exponentially [9,10], and it is still widely used nowadays. In addition to the health care industry or hospitals, other fields such as education [11], the transportation industry [12], banks [13], and international tourist hotels [14] also use DEA for performance evaluation.

In Taiwan, studies of medical institutions using DEA have included that by Chen, which applied the metafrontier analysis and Malquist index to assess the operational efficiency of 40 public hospitals and 79 private medical institutions with more than 100 hospital beds in 2019 [2]. The numbers of physicians, nursing staff, medical personnel, and hospital beds were used as input variables, and the number of outpatient and emergency visits, operations, and inpatient days were used as output variables. The three quality variables used were the number of net hospital deaths, referrals, and nosocomial infections. The findings revealed that the efficiency of public hospitals was higher than that of private hospitals. Ho (2017) adopted DEA and distinguished between different hospitals according to ownership as study subjects [3]. The results demonstrated that private hospitals had better efficiency and performance than public hospitals when categorized according to the different ownerships in Taiwan. In addition, scholars have used two-stage DEA to study the operational efficiency of 58 public hospitals and 124 private medical institutions in Taiwan with more than 50 beds, using the number of beds, administrative staff, physicians, nursing staff, and other medical personnel as input variables, and the number of hospitalizations, outpatients, annual emergency visits, inpatient income, and outpatient income as output variables. The results showed that in terms of technical efficiency, private hospitals performed better than public ones, and district hospitals performed better than medical centers and regional hospitals; in terms of scale efficiency (SE), public hospitals were more efficient than private hospitals, and medical centers were more efficient than district and regional hospitals. In the second stage of TOBIT regression, the hospital level, average inpatient stays, Herfindahl Hirschman index, and ownership were used as control variables. The findings revealed that hospitals with lower market competition and longer inpatient days were more efficient [4]. Kreng used two-stage DEA to evaluate the efficiency of the medical systems of 23 counties and cities under Taiwan’s single-payer system. Different from other studies, this study used counties and cities as DMUs, using the number of beds, full-time physicians, and full-time nursing staff as input variables; the number of outpatient visits, hospitalizations and emergency visits as intermediate variables; and outpatient income and inpatient income as output variables. The results revealed that Hsinchu and Taichung were the most inefficient, and the five cities with larger input also had larger output [5].

In Asia, there was a two-stage DEA study on the efficiency of medical systems in 46 Asian countries. Taking the average health care expenditure per person as the input variable, and the average active life expectancy and infant mortality rate as output variables, the findings revealed that 91.3% of the countries had inefficient medical systems, and the efficient countries were mostly high-income ones. There was an increasing return to scale (RS) of 39.1%, and a decreasing RS in 52.2% of the countries. In the TOBIT regression part, the number of physicians per thousand people, the number of beds per thousand people, the smoking rate of men, the number of residents per square meter, and the completion rate of primary education were used as control variables. The study concluded that the higher the completion rate of primary education, the better the country’s medical efficiency [15]. Another foreign study also included the evaluation of the efficiency value of 11 Palestinian public hospitals with two-stage DEA. The number of hospital beds, physicians, other medical professionals, and administrative staff were used as input variables, and the length of hospital stay, the number of outpatient and emergency visits were used as output variables. The findings showed under a fixed RS, the average efficiency value from 2010–2015 was 86%, which implied that the input could be reduced by 14% while maintaining the same output. However, the pure technical efficiency (PTE) under a variable RS was 93% on average, and large hospitals were less efficient than small ones. Moreover, taking the bed occupancy rate, the outpatient–inpatient rate, the inpatient days, the proportion of administrative medical personnel, the hospital size, the location, the proportion of refugees in the administrative area, the number of beds per 10,000 people in the administrative area, the number of care centers per 10,000 people in the administrative area, and the number of public beds in the administrative area as the TOBIT regression variables, it was concluded that the increase in the bed occupancy rate and the outpatient–inpatient rate enhance hospital efficiency, and the increase in the number of care centers per 10,000 people in the administrative area reduces hospital efficiency; furthermore, large hospitals are less efficient than small hospitals [16]. In the United States, some scholars also applied the Malmquist index and the investment-oriented DEA to evaluate the impact of the 2007–2008 financial crisis in Pennsylvania on the efficiency of 65 hospitals in the state. They used the number of hospital beds, the number of full-time physicians and dentists, the number of full-time nursing staff, and the county and city unemployment rate as input variables, and the number of inpatients, the inpatient days, the number of inpatient surgical operations, the total number of surgical operations, and the number of emergency visits as output variables. The study found that the financial crisis had an impact on hospital productivity, and the number of efficient hospitals increased gradually from 2005–2012. Furthermore, 15.4% of the hospitals were efficient in all years and 36.9% of the hospitals were inefficient in all years, and the lowest Malmquist index was indeed during the 2007 financial crisis [17].

The paper aimed to use the DEA and TOBIT regression to analyze the other factors that affect medical center efficiency, and different categories of hospitals are chosen to assess whether different years or different types of medical centers affect operational performance. This study provides reference values for the improvable directions of relevant large hospitals’ inefficiency decision-making units through reference group analysis and slack variable analysis.

## 2. Materials and Methods

### 2.1. Data Material

This is a retrospective panel-data study including all 19 tertiary hospitals in Taiwan. The data were obtained from open sources of government routine publications and hospitals disclosed by law to the NHI administration of Taiwan. The related variables included financial data from the public financial statements of the hospitals and the quality indicators from the online “NHI medical quality information disclosure network” [18]. All data were publicly available information; therefore, ethics approval and consent to data collection were not required. The collated period for data analysis was 2015–2018. Health statistics on the current status of medical institutions and hospitals’ medical service volume were from the Ministry of Welfare [19], and some demographic data were derived from the Global Information Network of the Household Registration, Ministry of the Interior, Taiwan [20].

### 2.2. DEA Methods

The basic models of DEA are two typical CCR and BCC models, which are also recognized in the literature as the two most influential models in the field [21]. CCR was jointly proposed by Charnes, Cooper and Rhodes in 1978, and calculates the overall efficiency of the organization assuming a fixed RS [22]. BCC is a modified CCR model with the addition of the concept of variable RS, which was proposed by Banker, Charnes and Cooper in 1984 [23], and it can derive a model that can measure pure technical efficiency, scale efficiency, and returns to scale [6]. It can derive a model that can evaluate the PTE, the SE, and the RS. Regardless of the CCR or BBC model, it can be divided into two orientations: the “input orientation” and the “output orientation”. For example, an input orientation discusses whether the input is efficient or not, whereas the output remains unchanged. This is because it is easier for hospitals to determine input variables than output variables. This research is based on the “input orientation” approach following other previous research papers of hospital [2,3,4,5] and NHI full coverage of the medical service environment in Taiwan, and the DEA model was developed prior to analysis. The CCR and BCC original math models of DEA were from Charnes, Cooper, Rhodes, and Banker et al. [22,23]. The performance of Taiwanese hospitals using models referring to the literature of Hsiao et al. [4] were assessed, and are shown in the formulas below. Let $x_{ij}$ denote the observed magnitude of *i* input for entity *j* ($x_{ij}$; *i* = 1,…, *m*; *j* = 1,…, *n*) and $y_{rj}$ denote the observed magnitude of *r* output for entity *j* ($y_{rj}\;$≥ 0; *r* = 1,…, *s*; *j* = 1,…, *n*).

$$\mathrm{CCR}:\;E_{k}=\mathrm{Maximize}=\frac{\sum_{r=1}^{s}u_{r}y_{rk}}{\sum_{i=1}^{m}v_{i}x_{ik}}\;\mathrm{Subject}\;\mathrm{to}\;\frac{\sum_{r=1}^{s}u_{r}y_{rj}}{\sum_{i=1}^{m}v_{i}x_{ij}}\leq 1,j=1,\ldots ,n;\;u_{r},v_{i}\geq \varepsilon ;r=1,\ldots ,s\;;\;i=1,\ldots ,m$$

where $v_{i}$, $r_{r}$ are the weights to be determined for input *i* and output *r*, and ε is a small positive value.

$$\mathrm{BCC}:\;E_{k}=\mathrm{Maximize}=\frac{\sum_{r=1}^{s}u_{r}y_{rk}-u_{0}}{\sum_{i=1}^{m}v_{i}x_{ik}}\;\mathrm{Subject}\;\mathrm{to}\;\frac{\sum_{r=1}^{s}u_{r}y_{rj}}{\sum_{i=1}^{m}v_{i}x_{ij}}\leq 1,j=1,\ldots ,N;\;u_{r},v_{i}\geq \varepsilon >0,\;j=1,\ldots ,n;\;r=1,\ldots ,s;\;i=1,\ldots ,m,$$

where ε is represented by a small non-Archimedean quantity, and $u_{0}$ is free in sign.

### 2.3. DEA Statistical Software/Analysis of Reference Groups & Difference Variables

At present, there are many types of DEA software, such as the Banxia Frontier Analyst, DEA Solver Pro, IDEAS, DEA Solver Online (DEAOS), DEAP2.1, and DEA Frontier. After evaluating the applicability of various software, this study adopted DEA Solver Pro V.15 as the analysis tool. For inefficient DMUs, DEA analysis can compare them with a similar but efficient unit to determine the reason for their inefficiency. Through the analysis of the reference group, a reference target for the relatively inefficient units is provided, and the relatively efficient units can also find out the number of times that they are referenced. Slack analysis represents the input that should be reduced or the output that should be increased by the relatively inefficient DMUs to achieve relative efficiency.

#### 2.3.1. DEA Input and Output Variable Selection

After completing the collection of variables, it was first confirmed that the number of variables was, at most, one-half of the DMUs according to the characteristics of DEA and, secondly, that there must be a positive relationship between the input and output variables. Subsequently, based on the analysis of correlation coefficients, the variables that were negatively correlated with other variables were eliminated, and the variables with higher positive correlations were selected as research variables [24,25]. In addition, as the variables cannot be negative when calculating DEA, the added value conversion method proposed by Pastor [26] was applied to add a fixed value to all variables with a negative value, so that there is no negative value in the variable. According to the characteristics of DEA, the research variables were divided into input and output variables. Based on the above-mentioned literature review and the expert opinions of the directors of the financial and operating units of Far Eastern Memorial Hospital, this study collected the appropriate input and output variables to construct its analysis model (Table 1).

#### 2.3.2. EBITDA Variable Selection

In the past, Earnings Per Share (EPS) was usually the basis for evaluating companies. However, as the health care industry in Taiwan is mostly non-profit and does not issue shares, EPS is not suitable for its evaluation. Apart from EPS, Earnings Before Interest, Taxes, Depreciation, and Amortization (EBITDA) is an alternative indicator, that is, the after-tax surplus adding back the amount of tax, interest, depreciation, and amortization. EBITDA was first proposed by John Malone, CEO of United States firm Tele-Communications Incorporated. He argued that taxes vary due to changes in regional or legal factors; therefore, it is not a factor that the company can control. Interest is a corporate financing means and has nothing to do with the firm’s profitability and operating ability. Depreciation and amortization are parts of the sunk cost; they are past costs which will not cause the expenditure of existing cash. After excluding the above-mentioned factors, EBITDA can reflect a company’s operating ability and is usually used to evaluate the value of the mergers and acquisitions industries. The main factor is that mergers and acquisitions allow companies to achieve economies of scale, using large-scale operations to share the depreciation expenses of non-cash expenditures, achieving a long-term profitable business strategy. Relying on a strong cash flow, companies can continue to carry out re-mergers. This is also one of the indicators that many investors pay attention to [27]. EBITDA can evaluate the cash flow aspect of a company, and in the health care industry, it can be used to assess the renewal capabilities of asset equipment and operational performance. In previous studies, EBITDA has mostly been used in the evaluation and credit of the business value and financial situation of an enterprise. In DEA-related studies, researchers have used EBITDA to evaluate the operational efficiency of cable and satellite companies and Internet channels [28,29]. However, there has yet to be research on the use of EBITDA for the efficiency analysis of hospitals. Due to Taiwan’s rising consumer price index, the cost of hospital construction 10 years ago is different from that 10 years later. Although Taiwan’s health insurance scheme has a fixed payment for every patient, the depreciation costs borne are different. Assume that two institutions have the same profit in the same year; however, the cash flows represented are completely different. Therefore, value, rather than profit, is the most important indicator used by the industry to evaluate an organization. Value presents the concept of using cash flow, that is, EBITDA. Although a hospital is a non-profit organization, it still needs to have good operating and financial performance. To fully reflect this assumption, this study adopted the revised EBITDA indicator in which current non-cash expenditure items, such as the depreciation for medical benefits are re-included, while other income from non-medical operations are excluded mainly because some consortium hospitals have high cash dividends. EBITDA was introduced in the discussion section on DEA in this study, in which the operating cash inflow ratio served as a measurement indicator. This indicator can also be used as an important indicator to test the capacity of a medical institution to make future business expansions, purchase new equipment, or survive a crisis. The originality of this study distinguishes itself from other previous studies [2,3,4,5].

The commonly used EBITDA indicator is medical profits plus depreciation and other non-cash expenditures, which is then divided by net income. However, as the simple numerical values in DEA studies better demonstrate the differences compared to the indicator categories, the EBITDA indicators and the EBITDA values were simultaneously collected as the research variable, and one of these was then selected as the final research variable through the correlation coefficient analysis. The EBITDA value is defined as medical profits plus non-cash expenditures, such as depreciation. In this study, to eliminate the pre-tax balance assessment bias caused by some medical centers with large dividends and non-operating revenues, the EBITDA value was revised to “medical profits plus depreciation and amortization”. This also eliminates the impact of non-cash depreciation expenditures on the operating results of the evaluation unit, and reflects the renewal capability of asset equipment and operational performance. That highlights the originality of this study compared to previous studies.

### 2.4. TOBIT Regression

Due to the particularity of DEA, the selection of input and output variables must comply with its premises. However, in addition to the selected input and output variables, there were also hospital characteristics, quality indicators, or environmental external factors to the hospital’s operational efficiency. Therefore, the statistical software SAS 9.4 was used to carry out the two-stage analysis of Tobit regression to help summarize other factors that affect hospital efficiency. This study used the efficiency value results of the CCR and BCC models as the dependent variables. As the efficiency value generated by DEA was between zero and one, which was a censored variable and unsuitable for the least square method estimation method of the general regression model, there may have been irrational phenomena which meant the estimated value was negative or greater than one. According to previous studies, Tobit regression is mostly used in coordination with DEA research. It was first published by Professor Tobit in 1958 [32], and was first applied in research by economist Goldberger in 1964 [33]. The TOBIT regression math model as follows, Y_*i*_ = β_0_ + β_1_X_*i*1_ + β_2_X_*i*2_ + … + β*_n_*X_*im*_ + ε. 0 ≤ Y_*i*_ ≤1; *i* = 1, 2…*n*; *m* =1, 2…*n*; Y_*i*_ = dependent variable; β_*n*_ = regression coefficient; X_*im*_ = the Xi DMUs, m’s independent variable; ε = residual.

#### TOBIT Regression Variable Selection

Tobit regression partly uses the efficiency values of CCR and BCC (i.e., overall efficiency, PTE) as the dependent variables to analyze other related factors that affect hospital efficiency. The study first selected 16 variables, collected data, and used different models for testing, and eventually selected seven research variables (Table 2). The variables selected in the two-stage TOBIT analysis were taken from previous studies [15,16,34]. Following correlation coefficient analysis, variables with lower correlations were excluded, and the first 16 variables were selected as follows: combination index CMI; hospital bed occupancy rate; medical profit ratio; the turnover rate of fixed assets; point value in different regions; nurse-to-patient ratio; number of physicians per 100,000 people; number of hospital beds per 100,000 people; number of hospital beds per 100,000 people; number of medical institutions in counties and cities; population density of counties and cities; population of counties and cities; population aged between 14 to 65 years; population aged above 65 years; ratio of population aged above 65 years in counties and cities; ratio of population aged below 14 years in counties and cities; and ratio of population aged below 14 years in counties and cities. Following discussions with experts, we excluded duplicate variables and adjusted several similar variables, and the seven variables listed in Table 2 were selected and included into the Tobit regression model for analysis. TOBIT Regression math model: Y_i_ = β_0_ + β_1 × 1i_ + β_2_X_2i_ + β_3_X_3i_ + β_4_X_4i_ + β_5_X_5i_ + β_6_X_6i_ + β_7_X_7i_ + ε.

Yi = the efficiency values of CCR (i.e., total efficiency, TE) and/or BCC (i.e., overall efficiency, PTE); β_0_ = residual.; β_1_ − β_n_ = regression coefficient of each variable; X_1_: The combination index CMI of inpatient record; X_2_: Hospital bed occupancy rate; X_3_: The turnover rate of fixed assets; X_4:_ Point value in different regions; X_5_: Number of medical staff per 100,000 people; X_6_: Number of hospital beds per 100,000 people; X_7_: The ratio of population over 65 and under 14 years; i = Number of hospitals; ε = residual.

## 3. Results

In this study, the findings indicate that different levels of operational efficiency and quality still exist in the tertiary hospitals of different types or attributes in Taiwan. The adoption of the DEA method and two-stage Tobit regression as analysis tools, as well as the innovative introduction of the application of the EBITDA indicator, offer different avenues of discussion and analysis to this research.

### Descriptive Statistics and Correlation Coefficient Analysis

The descriptive statistics analysis results are shown in Table 3. Although they were all tertiary medical centers in Taiwan, there were still differences in scale and service volume. Of the input variables, the total hospital beds (unit) had the largest coefficient of variation, followed by total physicians (person); of the output variables, the total relative value units (RVUs) for outpatient services (100 million NT$) had the largest coefficient of variation.

According to the DEA assumption, we should first confirm that the number of variables is, at most, one-half of the DMUs according to the characteristics of DEA. Secondly, there must be a positive relationship between the input and output variables [24,25]. This study was fitted with the conditions of the DEA method, but Lee and Choi (2010) proved that a cross-redundant output in a CCR or BCC DEA study is unnecessary and can be eliminated from the model without affecting the results of the study [35]. A cross-redundant output, as characterized by Lee and Choi, can be expressed as a specially constrained linear combination of both some outputs and some inputs. However, in the abstract of their article, Lee and Choi indicated that a cross-redundancy in DEA will occur “if a certain set of input and output variables is linearly dependent”. An example of cross-redundancy which they mention in their paper comes from the accounting field, where net income is revenue minus cost. Net income and revenue may be considered outputs in a DEA study, whereas cost may be an input [36]. In this study, the input and output variables were relatively independent variables, including correlations, but there is no relationship of mutual calculation between or within each other. Additionally, related researchers who can appropriately reduce the number of variables in the follow-up will get better results. There were 76 DMUs (19 hospitals × 4 years = 76) provided in this study, in which 11 variables (5 inputs and 6 outputs) were excluded. At the same time, we analyzed the correlation coefficients of the input and output variables, as shown in Table 4. In the correlation coefficient analysis, most of the input and output variables were highly correlated, among which the number of beds (Input-II) and the number of inpatient days (Output-IV) had the highest correlation coefficient of 0.988. In the input variables part, only the variables of the “emergency department transferred to hospitalization” and “staying in emergency department for more than 48 h”, which belonged to the quality category (Input-V), were relatively lowly correlated with each output variable, with the correlation coefficients falling within 0.147–0.359. In addition, the input variable of the equipment gross value (Input-IV) was also relatively lowly correlated with the overall output variables (Output-I) and the revised EBITDA value (Output-V), with correlation coefficients 0.33 and 0.372, respectively. Nonetheless, all input and output variables were still positively correlated (Table 4).

In the DEA relative efficiency analysis, Table 5 shows that in the overall efficiency value of CCR during four years, four hospitals (B, D, L, R) demonstrated efficiency (CCR = 1) for four consecutive years, which accounted for 21.05% (4 out of 19) of the 19 medical centers. Regarding BCC technical efficiency, eight hospitals (B, D, I, J, L, O, P, R) demonstrated efficiency for four consecutive years (BBC = 1), accounting for 42.10% (8 out of 19) of the 19 medical centers. Compared to CCR, there were four additional hospitals (I, J, O, P), which means that the inefficiency of the four hospitals might be caused by their size. Moreover, in terms of BCC SE and RS, the four above-mentioned hospitals (B, D, L, R) also showed good SE (SE = 1) for four consecutive years; in the three major medical centers in Taiwan (A, F, J), there were at least one to two decreasing, or two to three constant RS analysis results over the four-year period. This suggests that to continually pursue excellent health care quality, it is indeed difficult for the large-scale indicator-type medical centers in Taiwan to have a trend of an increasing RS.

In this section, we analyze the different categories in the ownership and region part. As medical centers of different attributes in Taiwan have different operational goals and corporate cultures, whether it is a public or private university-affiliated hospital, these hospitals possess higher teaching and research responsibilities, and the emphasis on operational performance is often weaker than that of the foundation hospitals donated to and invested in by large companies. The aim of the religion hospitals when setting up in Taiwan in the early days was mainly to serve the underprivileged, and there were often nongovernmental donations. Therefore, they do not advocate high self-pay health care services. Government hospitals are mostly large hospitals supported by central or local government budgets, and they have to go through more complicated procedures when purchasing expensive equipment (Table 6 and Table 7). Furthermore, the health care income of Taiwanese hospitals mainly comes from the NHI administration and less from the self-pay medical or healthcare services. As different regions belong to different health insurance branches (divided into six operation regions), the floating payment point value of each branch is different. Therefore, each of the hospital branches must be analyzed separately when evaluating the operational efficiency (Table 8). Overall, regardless of the attribute or region, efficiency values such as CCR (TE), BCC (PTE) and/or SE all demonstrated trends of continuous improvement over the four years from 2016–2018. This study analyzes the operating performance of the hospital for a total of 4 years. During this period, it may be due to the expansion of the new hospital or the impact of large-scale abnormal events (such as the COVID-19 pandemic). However, in the 2016–2018 period analyzed in this study, the research subjects (19 DMUs) did not have any major expansion or other events in Taiwan, so relevant confounder factors have been eliminated.

The categories of these four ownership types of registered hospitals are recognized by the Taiwanese government. Hospitals with the same ownership category have higher homogeneity. Regarding the results shown in Table 6, ownership was divided into four different groups, including university-affiliated hospitals, foundation hospitals, religion hospitals, and government hospitals. In terms of the average efficiency values of different attributes over the four years, government hospitals had the highest overall efficiency (TE) value in the CCR model, but there was no statistically significant difference between the four attributes. If considering the variable RS, the four-year average value of BCC (PTE) was highest for foundation hospitals, and the lowest was for government hospitals. This part reached a statistically significant difference (*p* = 0.007 **). This result aligns with the intuitive feelings of most people in Taiwan.

Regarding the results illustrated in Table 7, ownership was divided into two different groups, the private and the public hospitals. Table 7 suggests that if the attributes of all tertiary hospitals are only divided into two main categories, there are statistically significant differences in BCC PTE and SE; private hospitals demonstrated better BCC (PTE) than public hospitals (*p* = 0.021 *), whereas public hospitals showed better SE than private hospitals (*p* = 0.0036 ***). This result can be explained by the fact that public hospitals had more government budget support, thus the construction and scale of the hospitals may be better than that of private hospitals. However, private hospitals such as foundation hospitals or religion hospitals that emphasize operational performance had better PTE.

The results in Table 8 show that efficiency values such as CCR (TE), BCC (PTE), and/or SE demonstrated statistically significant differences in different regions. Nonetheless, in the PTC of BCC, the North and East regions both exhibited the highest efficiency (PTE = 1) over the four years. The main reason was that there was only one tertiary hospital in both regions (N = 1). In contrast, there were 42.1% (8 out of 19) medical centers in the region of North District City Center (N = 8) in Taiwan. Due to the fierce competition and large service volume, the health insurance floating payment point value of the region’s NHI administration branch would also be lower under the global budget payment system. Therefore, its CCR (TE) and SE were both ranked last in the six regions.

The results in Table 9 show the TOBIT regression coefficient and *p* values with the CCR (TE) and BCC (PTE) models; when the variables “The combination index CMI of inpatient record”, “Hospital bed occupancy rate”, and “The turnover rate of fixed assets” increase, the tertiary hospitals’ CCR (TE) increase. In addition, when the variables “The turnover rate of fixed assets” and “Number of hospital beds per 100,000 people” increase, the tertiary hospitals’ BCC (PET) also increase, but when the variables “The combination index CMI of inpatient record” and “Point value in different regions” increase, the BCC (PET) decrease. Because these variables have not been used in previous studies, we wanted to directly analyze and separately examine these medical institutions with different attributes and categories. Taiwan has a distinct healthcare context and NHI scheme. The scheme allocates different point values to hospitals based on location, operation category (university-affiliated hospitals, foundation hospitals, religion hospitals, and government hospitals), and ownership. Therefore, these variables were not included as the independent variables of the TOBIT regression.

## 4. Limitations

This study adopted DEA to analyze the operational efficiency of all tertiary hospitals in Taiwan. By adopting the two-step DEA procedure, we computed efficiency measurements in the first step and identified factors affecting efficiency in the second step. However, there are certain limitations in the time-based approach because the factors used in the second step may be critical to the construction of the efficiency frontier. Consequently, these factors affect both the distribution of efficiencies across DMUs and their size. Additionally, DEA is a deterministic non-parametric method. One of the disadvantages of DEA is the difficulty of applying statistical inference to DEA measurements. Instead, scholars have suggested implementing bootstrapping procedures to obtain more reliable and efficient measurements [37,38]. Because of time limit and budget constraints, there is no bootstrap of efficiency estimates of this study and no test to ascertain the separability between the sets of variables used in the DEA model and the variables used in the TOBIT regression. At the same time, the TOBIT regression was usually used to analyze the cross-sectional data, and this study used panel data, so for future research, the “Panel TOBIT Regression Model with Random Effects” could be analyzed. The following three research limitations existed in the process of data collection, which may have partially affected the research results: 1. Due to data source limitations, the calculated number of doctors, beds, and outpatient and inpatient medical expenses of the five large medical centers may have included those of their affiliated branches. 2. According to Article Four of the Measures for Financial Reporting by Medical Service Institutions of National Health Insurance Laws and Methods, Taiwan [39], medical institutions who receive insurance medical expenses exceeding a certain amount should submit a financial report to the NHI administration. However, as the financial report preparation regulations of health care institutions and public institutions are not the same, only two research variables could be chosen that could be compared with each other. 3. Although this study analyzed all the tertiary hospitals, a research sample size of 19 institutions is a minority, and so the results should be treated conservatively. Nevertheless, they are of reference value for Taiwan or other regions with systems similar to Taiwan’s NHI system. A majority of tertiary hospitals in other countries or medical centers in Taiwan must also assume social responsibilities and assist in promoting national health and welfare policies, insofar as promoting cross-border healthcare services. It is recommended that follow-up studies should include proxy outputs, such as a hospital‘s social responsibilities and the promotion of national health and welfare policies.

## 5. Discussions

Examining the relevant reference materials for applying DEA to health care or hospitals, for the effectiveness analysis of medical institutions, we analyzed the different attributes and other related factors on efficiency, such as regions, ownership, hospital type, bed occupancy rate, outpatient–inpatient ratio, hospital size, and primary health care centers. Most of the medical institutions in countries or regions to which the published literature belonged to had relatively simple attributes. Most studies in Taiwan have only compared public and private hospitals [2,3,4], or only assessed the effectiveness of government-operated public hospitals in different administrative regions [5], or only compared state-owned and private-owned hospitals out of 21 of the same level in 2007. In this study, the focus was on the operational efficiency of public hospitals in different administrative regions, rather than the operational efficiency of hospitals in different regions. Furthermore, one study [40] analyzed the data of a medical center only for the year 2007, whereas this study analyzed data across four consecutive years. However, the attributes of medical institutions in Taiwan are more diverse, and the insurance benefits paid by each administrative region of the NHI are different. Therefore, we added the “NHI point value in different regions” as a variable in the second stage of the TOBIT regression. Moreover, in addition to NHI payment, Taiwanese medical institutions emphasize non-NHI-related income sources, and hence, we also added “self-pay income“ as an output variable. Most of the above-mentioned published literature did not specifically limit the level and scale of the hospitals and did not distinguish between special hospitals’ categories in Taiwan. This study focused on hospitals in the same level (tertiary hospitals), collected data for four consecutive years, and increased the number of DMUs analyzed to 76. Therefore, in addition to eliminating the heterogeneity of medical institutions, we also increased the number of DMUs at the same time. Additionally, this study further explored Taiwanese medical institutions of different categories (see Table 6 and the footnote), and also analyzed the differences between public and private hospitals (see Table 7). Similar to previous studies, this study selected input or output variables and parameters for analysis by referring to the literature, thereby obtaining more robust analysis results.

The results of the research mostly showed that the performance of private hospitals was better than public ones. Under controlled conditions and research constraints, this study conducted an objective comparison between the single-level large teaching hospitals with different attributes. The findings reveal that the operational efficiency of Taiwan’s tertiary hospitals had different efficiency values in CCR (TE), BCC (PTE), and SE. Regardless of public or private hospitals, Taiwan’s largest hospitals (A, F, J) all had diminishing RS BCC. Meanwhile, as the public hospitals received more government budget support, the CCR (TE) was better than for private hospitals. Nevertheless, private hospitals, such as foundation hospitals or religion hospitals that paid more attention to operational performance, had better BCC (PTE) than public hospitals.

This study is different from the publications of DEA-related topics in other countries [16,41,42,43,44]. In the health care industry, there has been no prior literature including the EBITDA indicator of the general industry as DEA output variables to analyze the effectiveness of medical institutions. In this study, the EBITDA indicator of the general industry was converted into health care profits, using the depreciation expenditure as a research variable. In this manner, the deviation caused by the pre-tax balance evaluation of certain large foundation hospitals with huge dividends or non-industry income could be avoided, and the impact of sunk cost and depreciation expenditures could also be excluded to reflect the operational performance. Moreover, as the selection of the DEA input and output variables has certain limitations, to meet its premises, it was better to adopt a two-stage DEA when using it in a health care scenario. In addition to the analysis of the relative efficiency, TOBIT regression [4,42] was used for analysis in the second stage. This study also adopted the second stage of Tobit regression analysis, which only assisted in summarizing the input–output variables, but also helped to further understand whether there were other environmental or quality variables that affect the operational efficiency of Taiwan’s tertiary medical centers. The study findings reveal that the higher the bed occupancy rate and the fixed asset turnover rate, the higher the hospital CCR (TE); and the higher the fixed asset turnover rate and the number of beds per 100,000 people, the higher the BCC (PTE) of the medical center.

This study investigated the operational efficiencies of 19 medical centers in Taiwan using the DEA method and CCR and BCC model, and adopted the two-stage TOBIT regression model to further analyze and discuss the factors other than the input and output variables that also affect hospital efficiency. Unlike other studies that use commonly seen output variables such as inpatient expenses and inpatient days [2,45], this research innovatively adds the relatively rare modified EBITDA indicator to the output variables in the analysis of healthcare DEA. The study findings show that the two indicators of “modified EBITDA” and “self-pay income” in the output variables had a certain degree of discrimination as the output indicators of financial efficiency, and could more easily distinguish the operation efficiencies between foundation hospitals or compare their operational benefits to hospitals with different attributes. A research limitation of this study is that the operational information or financial statements required for the research could, to a certain extent, be regarded as confidential hospital information. Therefore, unless public disclosure was legally required, it was difficult to obtain. Even if public information is used, there are certain research limitations due to the inconsistency of the data format by different ownership hospitals, making it hard to compare. Hence, if researchers wish to conduct future in-depth research, they may consider applying for undisclosed government databases or the more realistic financial statements and operational data of hospitals. By excluding certain errors caused by second-hand data and the limitations of public data, researchers should be able to produce more accurate research and analysis reports. 

## 6. Conclusions

Under the universal coverage of the Taiwanese NHI, hospitals are facing rising costs and pressures on resources to meet budget constraints. Health care managers face various challenges in the highly competitive environment of Taiwan. The results of this research demonstrate that the relatively large tertiary hospitals were more likely to experience diminishing RS, and it was recommended to adjust their economic scale. Moreover, the study findings indicate that 57.8% (11 out of 19) of medical centers were inefficient in providing health services.

At present, the main health insurance systems in the world are the Beveridge Model, Bismarck Model, Single Payer System, and Pocket Model. Taiwan adopts the Single Payer System, where the government mandates that people are insured, and that they pay different health insurance fees based on their income. Medical institutions provide medical services to the people and then apply for payment to the NHI administration. NHI adopts a global budget payment system and appropriately claims the point value of the health insurance benefits in Taiwan. However, due to Taiwan’s more special medical service environment, there are more research limitations in this study, only for further reference by researchers in Taiwan or researchers in countries with special medical insurance systems similar to Taiwan’s medical environment, because the different environment may not be applicable to other relevant medical institutions DEA analysis.

The findings of this study can provide health care administrators and hospital managers with a reference for allocating resources in order to achieve high efficiency in Taiwan; also, the results of this study can serve as a reference for improving the operational efficiency of medical centers in Taiwan and provide indicators that were not previously used in Taiwan medical institutions to facilitate the self-examination of their operating performance. Additionally, the results and recommendations of this study can be used as a reference for medical centers in Taiwan to determine what kind of resources should be invested to produce better operational profit and gain more benefits.

This study finally compared with recent references which reviewed 262 papers on DEA applications in healthcare, with a special focus on hospitals which has been published between 2005 to 2016; this review paper analyzed the DEA methodological settings and described the new applied models in the healthcare system [46]. Each of the input and output variable numbers around three to five were typical selection models, due to the differences in medical insurance and healthcare service environments in different countries. This study summarized the variables used in the published references in Taiwan, and the five input and six output variables used were selected. Most of them also belong to the first few important variables used after this reference was compiled into multiple journal papers, but whether it is appropriate to reduce the number of variables is still worth discussing. Furthermore, reducing the number of input and output variables in the first analysis and adding the test for separation in the second stage of DEA will improve the study quality and avoid bias in this study. However, the variables of this study are all variables that belong to the Taiwanese healthcare service environment that is of great concern to hospital managers, and a special variable (EBITDA) that this study hopes to present is specially selected, where such research results still have a certain reference value in Taiwan but still have to verified further. The preliminary results of this study will provide follow-up researchers with the optional references for reducing variables, and will also provide a direction of further analysis after this study.

## Figures and Tables

**Table 1:** The list of study input and output DEA variables.

Variable		Variable Definition
Input variables	total physicians [2,4,5,16]	The total number of Western and Chinese medicine practitioners and dentists in the latest hospital practice registration in the statistics file of the medical personnel category in the health care management subsystem.
	total hospital beds [4,5,16,30]	The total number of hospital beds, including emergency room beds, hemodialysis beds, nursery beds, obstetric wards, other observation beds, peritoneal dialysis beds, and so forth.
	fixed assets net value [30]	The net fixed assets items in the public financial statement of each hospital.
	gross equipment [31]	The gross amount of machinery and equipment in the balance sheet for public hospitals; the gross amount of medical equipment in the balance sheet for private hospitals.
	the rate of emergency transfer in-patient stay over 48 h [2,30] *	(Number of cases with >48 h in the emergency department/number of cases transferred from emergency department to admission) × 100%.
Output variables	surplus or deficit of appropriation ^#^	For public hospitals, it is the value of the remaining (short) items in the income and expenditure balance sheet of the current period; for private hospitals, it is the value of the after-tax items in the income and expenditure balance sheet of the current period.
	the total RVUs for outpatient services [4,5]	The total number of acute bed days and chronic bed days in each hospital in the 2nd generation storage and inpatient detail files of the NHI administration, except for when the declaration field “Not applicable to Taiwan Diagnosis Related Groups (Tw-DRGs) Case Special Note” reads “9: Cases of declared cut accounts that have not been discharged within 30 days of hospitalization”, which is not included in the calculation.
	the total RVUs for inpatient services [4,5]	All outpatient medical expenses declared by the hospital (ex. Western medicine, Chinese medicine, dentist, dialysis, etc.), including application points and copayments in the second-generation storage and admission detail files of the NHI administration.
	length of stays [2,4,16]	All inpatient medical expenses declared by the hospital (ex. Western medicine, Chinese medicine, dentist, dialysis, etc.) in the second-generation storage and admission detail files of the NHI administration.
	modified EBITDA ^#^	The health care profits of each hospital adding back the expenditures of depreciation and amortization.
	self-pay income ^#^	For public hospitals, this is defined as [medical income–(medical expenses in the report of the hospitals’ health care service declaration status of the National Health Insurance Administration × regional point value)]; for private hospitals, it is defined as the non-health insurance income in detail files of health care income.

Note: Numbers in brackets denote the relevant studies that support the use of a variable; studies marked with an asterisk (*) have used medical quality-related variables (variables related to the quality measures of process evaluation); variables marked with a hashtag (^#^) have not been used in literature; the calculation of “surplus or deficit of appropriation” is explained as follows: Because the operating costs of a tertiary hospital includes teaching and research expenses, the diminishment of this variable can be partially attributed to the tertiary hospital’s increased teaching and research expenses or duty to treat patients with more severe illnesses. Those variables were from previous related studies in Taiwan; the selection of inputs and outputs variables as well as the internal management factors might need some discussion argumentative backing in different countries.

**Table 2:** A list of studies of other TOBIT regression variables.

**Variables**		**Variable Definition**
Other variables	The combination index CMI of inpatient record [34]	Σ (Number of cases per DRG x relative weight of each DRG)/(The total number of cases in all DRGs)
	Hospital bed occupancy rate [16]	The number of occupied days declared for various types of beds/(the number of occupied days of beds declared by the hospital * number of beds in the month)
	The turnover rate of fixed assets [30]	Gross income on each hospital’s financial statement/net fixed assets
	Point value in different regions ^#^	Calculated using the average point value of the health insurance zone of the hospital
	Number of medical staff per 100,000 people [15,16]	The number of medical personnel per 100,000 people in the county or city where the medical center is located.
	Number of hospital beds per 100,000 people [15]	The number of hospital beds per 100,000 people in the county or city where the medical center is located.
	The ratio of population over 65 and under 14 year [15] *	Population over 65 years old and under 14 years old in the county or city where the medical center is located/total population.

Note: Numbers in brackets denote the relevant studies that support the use of a variable; studies marked with an asterisk (*) have used the age-related variable (% of relevant age group); variables marked with a hashtag (^#^) have not been used in literature; CMI: Case Mix Index, a measure that reflects the diversity, complexity, and severity of patient illnesses treated at a given hospital. It means the higher the CMI, the higher the responsibility of the hospital in teaching and research and treating severe illness.

**Table 3:** Descriptive statistics results of input and output variables.

Variables		Max	Min	$\overset{-}{X}$	SD	CV
Input variables	total physicians (person)	1723	357	780.18	360.17	2.166
	total hospital beds (unit)	3665	725	1688.42	777.38	2.172
	fixed assets net value (100 million NT$)	305.71	5.40	84.59	68.99	1.226
	gross equipment (100 million NT$)	116.85	13.46	41.83	22.83	1.832
	the rate of emergency transfer in-patient stay over 48 h (%)	0.27	0.00	0.07	0.06	1.167
Output variables	surplus or deficit of appropriation, after value-added conversion (100 million NT$)	79.86	0.00	10.48	16.10	0.651
	the total relative value units (RVUs) for outpatient services (100 million NT$)	125.24	21.69	56.13	28.11	1.997
	the total relative value units (RVUs) for inpatient services (100 million NT$)	113.16	15.15	47.41	25.30	1.874
	length of stays (10,000 days)	106.27	16.92	48.96	24.77	1.977
	modified EBITDA, after value-added conversion (100 million NT$)	31.10	0.00	9.82	7.61	1.290
	self-pay income (100 million NT$)	73.67	8.30	26.95	15.24	1.768

Note: Max: maximum value; Min: minimum value; $\overset{-}{X}$: mean; SD: standard deviation; CV: coefficient of variation. DMUs = 19 × 4, N = 76.

**Table 4:** Correlation coefficient analysis table of variables.

Variables		Input Variables					Output Variables					
		Input-I	Input-II	Input-III	Input-IV	Input-V	Output-I	Output-II	Output-III	Output-IV	Output-V	Output-VI
Input variables	I: Total physicians	1	0.940 **	0.833 **	0.733 **	0.315 *	0.711 **	0.967 *	0.973 **	0.947 **	0.695 **	0.922 **
	II: Total hospital beds	0.940 **	1	0.833 **	0.675 **	0.186	0.739 **	0.916 **	0.964 **	0.988 **	0.647 **	0.894 **
	III: Fixed assets net value	0.833 **	0.833 **	1	0.732 **	0.277 *	0.683 **	0.837 **	0.856 **	0.837 **	0.493 **	0.857 **
	IV: Gross equipment	0.733 **	0.675 **	0.732 **	1	0.235 *	0.330 *	0.711 **	0.750 **	0.706 **	0.372 *	0.651 **
	V: Rate of emergency transfer in-patient stay over 48 h	0.315 *	0.186	0.277 *	0.235 *	1	0.147	0.320 *	0.252 *	0.231 *	0.359 *	0.268 *
Output variables	I: Surplus or deficit of appropriation	0.711 **	0.739 **	0.683 **	0.330 *	0.147	1	0.719 **	0.745 **	0.718 **	0.521 **	0.761 **
	II: Total relative value units (RVUs) for outpatient services	0.967 **	0.916 **	0.837 **	0.711 **	0.320 *	0.719 **	1	0.967 **	0.930 **	0.764 **	0.941 **
	III: Total relative value units (RVUs) for inpatient services	0.973 **	0.964 **	0.856 **	0.750 **	0.252 *	0.745 **	0.967 **	1	0.972 **	0.698 **	0.930 **
	IV: Length of stays	0.947 **	0.988 **	0.837 **	0.706 **	0.231 *	0.718 **	0.930 **	0.972 **	1	0.646 **	0.880 **
	V: Modified EBITDA	0.695 **	0.647 **	0.493 **	0.372 *	0.359 *	0.521 **	0.764 **	0.698 **	0.646 **	1	0.782 **
	VI: Self-pay income	0.922 **	0.894 **	0.857 **	0.651 **	0.268 *	0.761 **	0.941 **	0.930 **	0.880 **	0.782 **	1

Note: * *p* < 0.05; ** *p* < 0.01; Input-I: total physicians; Input-II: total hospital beds; Input-III: fixed assets net value; Input-IV: gross equipment; Input-V: the rate of emergency transfer in-patient stay over 48 h; Output-I: surplus or deficit of appropriation; Output-II: the total relative value units (RVUs) for outpatient services; Output-III: the total RVUs for inpatient services; Output-IV: length of stays; Output-V: modified EBITDA; Output-VI: self-pay income. Grey part: the background color part represents the symmetrical correlation index.

**Table 5:** The DEA relative efficiency value of each tertiary hospital in Taiwan.

DMUs	2015				2016				2017				2018			
	CCR	BCC	SE	RS	CCR	BCC	SE	RS	CCR	BCC	SE	RS	CCR	BCC	SE	RS
A *	0.950	0.959	0.990	Decreasing	1	1	1	Constant	0.994	1	0.994	Decreasing	1	1	1	Constant
B	1	1	1	Constant	1	1	1	Constant	1	1	1	Constant	1	1	1	Constant
C	0.843	1	0.843	Increasing	0.787	0.982	0.801	Increasing	0.771	1	0.771	Increasing	0.771	0.986	0.782	Increasing
D	1	1	1	Constant	1	1	1	Constant	1	1	1	Constant	1	1	1	Constant
E	0.959	0.959	1	Constant	0.998	0.998	1	Constant	1	1	1	Constant	1	1	1	Constant
F *	0.951	0.957	0.994	Constant	1	1	1	Constant	0.952	0.998	0.953	Decreasing	1	1	1	Constant
G	0.954	0.972	0.982	Increasing	0.967	0.988	0.978	Increasing	0.989	1	0.989	Increasing	1	1	1	Constant
H	0.915	1	0.915	Increasing	0.936	0.991	0.944	Increasing	1	1	1	Constant	1	1	1	Constant
I	1	1	1	Constant	0.973	1	0.973	Increasing	0.971	1	0.971	Increasing	1	1	1	Constant
J *	0.988	1	0.988	Decreasing	0.968	1	0.968	Decreasing	1	1	1	Constant	1	1	1	Constant
K	1	1	1	Constant	1	1	1	Constant	0.880	0.883	0.996	Increasing	1	1	1	Constant
L	1	1	1	Constant	1	1	1	Constant	1	1	1	Constant	1	1	1	Constant
M	0.903	0.926	0.975	Increasing	0.914	0.945	0.968	Increasing	0.917	0.951	0.965	Increasing	0.901	0.948	0.950	Increasing
N	0.997	1	0.997	Increasing	1	1	1	Constant	0.994	0.996	0.998	Constant	1	1	1	Constant
O	0.755	1	0.755	Increasing	0.834	1	0.834	Increasing	0.954	1	0.954	Increasing	0.998	1	0.998	Increasing
P	0.947	1	0.947	Increasing	0.946	1	0.946	Increasing	0.985	1	0.985	Increasing	1	1	1	Constant
Q	0.840	0.997	0.842	Increasing	0.878	1	0.878	Increasing	0.937	1	0.937	Increasing	1	1	1	Constant
R	1	1	1	Constant	1	1	1	Constant	1	1	1	Constant	1	1	1	Constant
S	0.892	0.976	0.914	Increasing	0.897	0.942	0.952	Increasing	1	1	1	Constant	0.958	0.993	0.964	Increasing

Note: * The three major medical centers with the highest number of beds, services, and public confidence. DMUs: decision-making units, total DMUs = 76 = 19 (the codes of different hospitals A to S) times 4 years (2015–2018); CCR: overall efficiency value, total efficiency (TE); BCC: pure technical efficiency value (PTE), SE: scale efficiency (PTE); RS: returns to scale.3.2. DEA average efficiency value with different categories of hospitals.

**Table 6:** The DEA average efficiency value with different categories of hospitals.

Ownership	Years	CCR (TE) (*p* = 0.861)		BCC (PTE) (*p* = 0.007 *)		SE (*p* = 0.2644)	
University-affiliated Hospitals # N = 5 × 4 (DMUs)	2015	$\overset{-}{X}$*_i_* 0.951	0.949	$\overset{-}{X}$*_i_* 0.994	0.986	$\overset{-}{X}$*_i_* 0.956	0.962
	2016		0.951		0.994		0.956
	2017		0.950		0.999		0.950
	2018		0.954		0.997		0.957
Foundation Hospitals N = 7 × 4 (DMUs)	2015	$\overset{-}{X}$*_i_* 0.962	0.928	$\overset{-}{X}$*_i_* 1	1	$\overset{-}{X}$*_i_* 0.963	0.929
	2016		0.941		0.999		0.943
	2017		0.980		1		0.980
	2018		1		1		1
Religion Hospitals N = 3 × 4 (DMUs)	2015	$\overset{-}{X}$*_i_* 0.959	0.946	$\overset{-}{X}$*_i_* 0.983	0.992	$\overset{-}{X}$*_i_* 0.975	0.954
	2016		0.947		0.981		0.966
	2017		0.955		0.961		0.994
	2018		0.986		0.998		0.988
Government Hospitals N = 4 × 4 (DMUs)	2015	$\overset{-}{X}$*_i_* 0.968	0.953	$\overset{-}{X}$*_i_* 0.980	0.960	$\overset{-}{X}$*_i_* 0.988	0.993
	2016		0.978		0.986		0.992
	2017		0.967		0.987		0.980
	2018		0.975		0.987		0.988

Note: The categories of these four ownership types of registered hospitals are recognized by the Taiwanese government; # University-affiliated hospitals were including public and private hospitals; the government hospitals were all public hospitals, foundation and religion hospital were all private hospitals. TE: Total Efficiency; PTE: Pure Technical Efficiency; SE: Scale Efficiency; *p*: *p*-value; *: *p* < 0.05; $\overset{-}{X}$*_i_*: the mean value for the period from 2016 to 2018.

**Table 7:** The DEA average efficiency value of private and public tertiary hospitals.

Ownership	Years	CCR (TE) (*p* = 0.127)		BCC (PTE) (*p* = 0.021 *)		SE (*p* = 0.0036 *)	
Private Hospitals N = 13 × 4 (DMUs)	2015	$\overset{-}{X}$*_i_* 0.954	0.937	$\overset{-}{X}$*_i_* 0.995	0.998	$\overset{-}{X}$*_i_* 0.959	0.939
	2016		0.940		0.993		0.946
	2017		0.961		0.991		0.970
	2018		0.979		0.998		0.981
Public Hospitals N = 6 × 4 (DMUs)	2015	$\overset{-}{X}$*_i_* 0.973	0.953	$\overset{-}{X}$*_i_* 0.983	0.962	$\overset{-}{X}$*_i_* 0.989	0.990
	2016		0.980		0.989		0.991
	2017		0.975		0.991		0.984
	2018		0.983		0.991		0.992

Note: TE: Total Efficiency; PTE: Pure Technical Efficiency; SE: Scale Efficiency; *p*: *p*-value; *: *p* < 0.05; $\overset{-}{X}$*_i_*: the mean value for the period from 2016 to 2018.

**Table 8:** The DEA average efficiency value with different region tertiary hospitals.

Region	Years	CCR (TE) (*p* = 0.315)		BCC (PTE) (*p* = 0.388)		SE (*p* = 0.1362)	
North District City Center N = 8 × 4 (DMUs)	2015	$\overset{-}{X}$*_i_* 0.962	0.926	$\overset{-}{X}$*_i_* 0.993	0.989	$\overset{-}{X}$*_i_* 0.968	0.937
	2016		0.956		0.999		0.957
	2017		0.965		0.985		0.979
	2018		1		1		1
North N = 1 × 4 (DMUs)	2015	$\overset{-}{X}$*_i_* 0.989	0.988	$\overset{-}{X}$*_i_* 1	1	$\overset{-}{X}$*_i_* 0.989	0.988
	2016		0.968		1		0.968
	2017		1		1		1
	2018		1		1		1
Center N = 4 × 4 (DMUs)	2015	$\overset{-}{X}$*_i_* 0.930	0.924	$\overset{-}{X}$*_i_* 0.990	0.984	$\overset{-}{X}$*_i_* 0.939	0.939
	2016		0.920		0.980		0.939
	2017		0.943		1		0.943
	2018		0.932		0.995		0.937
South N = 2 × 4 (DMUs)	2015	$\overset{-}{X}$*_i_* 0.982	0.977	$\overset{-}{X}$*_i_* 0.995	0.986	$\overset{-}{X}$*_i_* 0.987	0.991
	2016		0.970		0.994		0.976
	2017		0.980		1		0.980
	2018		1		1		1
South District City Center N = 3 × 4 (DMUs)	2015	$\overset{-}{X}$*_i_* 0.969	0.967	$\overset{-}{X}$*_i_* 0.980	0.975	$\overset{-}{X}$*_i_* 0.988	0.991
	2016		0.971		0.982		0.990
	2017		0.970		0.982		0.988
	2018		0.967		0.983		0.984
East N = 1 × 4 (DMUs)	2015	$\overset{-}{X}$*_i_* 0.969	0.947	$\overset{-}{X}$*_i_* 1	1	$\overset{-}{X}$*_i_* 0.969	0.947
	2016		0.946		1		0.946
	2017		0.985		1		0.985
	2018		1		1		1

Note: TE: Total Efficiency; PTE: Pure Technical Efficiency; SE: Scale Efficiency; *p*: *p*-value; $\overset{-}{X}$*_i_*: the mean value for the period from 2016 to 2018.

**Table 9:** The TOBIT regression results of the DEA CCR and BCC models.

TOBIT Regression	CCR Model (TE)		BCC Model (PET)	
Variables	regression coefficient	*p*-value	regression coefficient	*p*-value
Regression intercept	0.605	0.564	4.390 *	0.0001
The combination index CMI of inpatient record	0.227 *	0.018	−0.108	0.228
Hospital bed occupancy rate	0.529 *	<0001	0.144	0.163
The turnover rate of fixed assets	0.028 *	0.001	0.020 *	0.046
Point value in different regions	−0.442	0.633	−3.869 *	0.020
Number of medical staff per 100,000 people	0.005	0.471	−0.048 *	0.044
Number of hospital beds per 100,000 people	−0.006	0.406	0.100 *	0.040
The ratio of population over 65 and under 14 year	0.036	0.975	0.213	0.805

Note: TE: Total Efficiency; PTE: Pure Technical Efficiency; *: *p*-value < 0.05.

## Data Availability

The data for this research was sourced from open government information, and hospitals by law required disclosing to the National Health Insurance Administration, Ministry of Health and Welfare.
